# Acetylated, phosphorylated and sulfonated nanocellulose for the modification of urea-formaldehyde adhesive in particleboard production

**DOI:** 10.1038/s41598-026-56716-x

**Published:** 2026-06-08

**Authors:** Jakub Kawalerczyk, Dorota Dziurka, Magdalena Woźniak, Dorota Dukarska, Radosław Mirski

**Affiliations:** 1 Department of Mechanical Wood Technology, Faculty of Forestry and Wood Technology, Poznan University of Life Sciences, Wojska Polskiego 38/42, Poznań, 60-627 Poland; 2 Department of Chemistry, Faculty of Forestry and Wood Technology, Poznan University of Life Sciences, Wojska Polskiego 75, Poznań, 60-625 Poland

**Keywords:** Nanocellulose, Urea-formaldehyde resin, Particleboard, Acetylation, Phosphorylation, Sulfonation, Chemistry, Materials science

## Abstract

The functionalization of nanocellulose (NC) to enhance its effect on UF resin properties and wood-based materials still remains insufficiently explored. NC was modified via acetylation, phosphorylation, and sulfonation. ATR-FTIR analysis combined with quantitative methods, including acid-base titration, potentiometric titration, and elemental analysis, confirmed successful NC functionalization. Both unmodified and modified NC affected the properties of urea-formaldehyde (UF) adhesive mixtures. Gel time decreased in adhesives containing acetylated NC, while viscosity increased most in mixtures with phosphorylated and sulfonated NC. Thermogravimetric analysis of UF adhesives containing NC showed improved stability, with phosphorylated NC providing the highest thermal resistance and acetylated NC enhancing stability only at lower temperatures up to 250 °C. Mechanical testing indicated that the incorporation of NC enhanced the strength of the particleboards. Variant containing acetylated NC achieved the highest internal bond and was the only one among functionalized variants to significantly improve bending strength and modulus of elasticity. Dimensional stability was enhanced, with acetylated NC reducing thickness swelling after both short- and long-term water exposure and limiting water absorption after short-term exposure. Phosphorylated and sulfonated NC also reduced thickness swelling, but only during short-term exposure. Overall, acetylation was the most effective modification, enhancing UF adhesive performance and particleboard quality in the most significant way.

**Supplementary Information:**

The online version contains supplementary material available at 10.1038/s41598-026-56716-x.

## Introduction

Cellulose, the most abundant biopolymer on Earth and a primary structural component of plant cell walls, has a complex structure with numerous inter- and intramolecular hydrogen bonds, leading to a high abundance of hydroxyl groups along the polymer chain^1^. Advances in materials science have enabled the isolation of cellulose at the nanoscale, where at least one dimension is smaller than 100 nm, using methods such as, for example, acid hydrolysis, oxidation, mechanical treatment, enzymatic hydrolysis, solvent-based techniques, or their combinations^2^. Significant advances in nanocellulose research over recent years have led to growing interest in its potential uses. As a result, the global NC market was valued at USD 319.5 million in 2021 and is projected to reach USD 1.063 billion by 2028, with a compound annual growth rate (CAGR) of 22.2%. This rapid growth has driven increased involvement from both startups and established companies in its production and commercialization^3^. NC is being explored for a wide range of applications across various industries, including its use to improve the properties of wood-based materials. The vast majority of available studies focus on incorporating cellulosic nanoparticles into resins used as binders, especially urea-formaldehyde (UF) resin. It has been demonstrated that the addition of small amounts of NC to UF resin contributes to the improvement of properties in wood-based materials such as particleboard^4^, oriented strand board (OSB)^5^, plywood^6^, laminated veneer lumber (LVL)^7^, and medium density fiberboard (MDF)^8^. However, the potential of NC can be further enhanced through its modification.

The chemistry of NC provides great potential as the abundant hydroxyl groups can be substituted with various functional groups to create diverse derivatives^9^. This is particularly important considering that certain drawbacks, such as agglomeration and limited compatibility with polymeric matrices, may limit the overall potential of NC in various applications, including its use as a modifier of UF resin. Fortunately, as shown by previous studies, these challenges can be at least partially mitigated through appropriate chemical modification^10^. Studies have shown that amination of nanocellulose using 3-aminopropyltriethoxysilane (APTES) or ethylenediamine (EDA) improved the physical and mechanical properties of wood-based materials. Moreover, due to the attachment of amino groups, these modifications also enhanced the formaldehyde-scavenging effect of NC^11–14^. Studies have also demonstrated that modification of NC with 4,4′-diphenylmethane diisocyanate (MDI) prior to its addition to UF resin improved the strength and water resistance of the resulting particleboards^15^. However, numerous methods such as, for example, acetylation, phosphorylation, and sulfonation, frequently discussed in review articles on nanocellulose functionalization^16–20^, have not been investigated in the area of formaldehyde-based resins modification with nanoparticles and their use for wood-based materials production so far.

According to Zhou et al.^21^, acetylation is a well-established method for modifying nanocellulose, where acetyl groups are attached to its structure by reacting with its hydroxyl groups. It usually leads to improved compatibility with various polymer matrices. The use of acetylated nanocellulose has been investigated in studies focusing, among others, on polycaprolactone^22^, bio-inks^23^, poly(ethylene-co-vinyl acetate) foams^24^, polylactide^25^, nitrile butadiene rubber^26^, films for food packing^27^, polypropylene^28^, hydroxypropyl starch acetate^29^, acrylic resin^30^, and epoxy resin^31^. According to Patoary et al.^32^, phosphorylation of NC is the process of adding phosphate moieties to the cellulose backbone, which is as an important route for producing diverse phosphate derivatives of cellulosic functional materials. The application of phosphorylated nanocellulose has been investigated in various materials, including, among others, nitrile butadiene rubber^33^, polyethylene^34^, starch films^35^, polyvinyl alcohol membranes^36^, chitosan films^37^, and epoxy films^38^. Sulfonation is a chemical modification process that introduces sulfonic functionalities onto NC surface^39^. Sulfonated NC was previously used in chitosan composites^40^, transparent films^41^, polyacrylamide sensors^42^, polylactide^43^, polyaniline membranes^44^, and epoxy coatings^45^. The reported improvements in nanoparticles dispersion and compatibility with various matrices resulting from these chemical modifications indicate their strong potential to enhance the performance of both UF resin and the particleboards, particularly in terms of internal bond strength, flexural properties, and water resistance, which are crucial for wood-based materials intended for indoor applications.

Considering the potential to enhance the effect of NC addition to UF resin on the properties of wood-based materials through its functionalization, the encouraging results reported for the planned modification methods, and the lack of knowledge about their application in related research, further studies are needed. Therefore, the initial hypothesis was that acetylation, phosphorylation, and sulfonation of NC prior to incorporation into UF resin will significantly improve the mechanical (internal bond, bending strength and modulus of elasticity) and physical (thickness swelling and water absorption) properties of the resulting particleboards.

## Materials and methods

### Materials

A re-dispersible dry powder of nanocrystalline cellulose (NC), obtained via sulphuric acid hydrolysis, was purchased from Nanografi Laboratory (Ankara, Türkiye). According to transmission electron microscopy (TEM) images provided by the manufacturer, the powder contained nanoparticles with widths ranging from 10 to 20 nm and lengths between 300 and 900 nm. Based on the technical data, the NC was characterized by the presence of hydroxyl groups as the main functional groups, a bulk density ranging between 0.5 and 0.8 g/cm^3^, a moisture content of approximately 4%, a crystallinity index of 92%, and a decomposition temperature of 349 °C. UF resin and industrial pine (*Pinus sylvestris* L.) wood particles were supplied by a local wood-based materials manufacturer. The resin was characterized by a viscosity of 254 mPa·s, a solid content of 59–61%, a gel time of 126 s at 100 °C, and a pH of 7.5. The wood particles were characterized using a sieve analysis repeated three times and measurements of 300 individual particles to determine their average size and shape. The results of the particles’ geometry analysis are provided in the supplementary materials (Figure S1, Table S1). All reagents, including acetic anhydride (99.5%, Sigma-Aldrich), acetone (analytical grade, Stanlab), acetylacetone (> 99%, Chempur), ammonium acetate (analytical grade, Chempur), ammonium nitrate (analytical grade, Chempur), ethanol (96%, Poch), potassium chloride (> 99%, Sigma-Aldrich), sodium hydroxide (> 99%, Sigma-Aldrich), phosphoryl chloride (99%, Sigma-Aldrich), sodium bisulfite (99%, Sigma-Aldrich), sodium periodate (> 99.8%, Sigma-Aldrich), sulfuric acid (98%, Sigma-Aldrich), toluene (> 99.5%, Poch), tetrahydrofuran (> 99.9%, Sigma-Aldrich), and hydrochloric acid (analytical grade, Poch), were used as received without further purification.

### Nanocellulose modification

Acetylation of NC was carried out using acetic anhydride under the conditions identified by Thien et al.^46^ as the most favourable among various reaction temperatures, reaction times, and NC-to-acetic anhydride ratios. The reaction was performed in a 500 ml flask equipped with a magnetic stirrer, reflux condenser, and thermometer. A total of 2 ± 0.001 g of NC was dispersed in acetic anhydride, and 1.6 ml of 98% sulfuric acid was added as a catalyst. The mixture was reacted at 70 °C for 90 min, using a NC-to-acetic anhydride ratio of 1:15 (w/v). After the reaction, the mixture was cooled, centrifuged at 6000 rpm (corresponding to approx. 3100 × g) for 8 min using an IFuge D06 Premium Edition centrifuge (JP Selecta, Barcelona, Spain), and the NC was washed with ethanol and acetone to remove any unreacted acetic anhydride, following the procedure recommended by Zimmermann et al.^24^. Then, the NC was subjected to several cycles of washing with distilled water, centrifugation, and dried at 60 °C for 24 h. The resulting material was labeled as NC-A.

Phosphorylation was carried out using phosphoryl chloride according to the method described by Blilid et al.^37^. A total of 2 ± 0.001 g of NC was dispersed in 50 ml of tetrahydrofuran and stirred for 10 min at room temperature. Then, 10 ml of phosphoryl chloride was slowly added to the suspension under continuous stirring. The reaction mixture was stirred at room temperature for 24 h. Then, 10 ml of deionized water was introduced into the mixture and stirred for 1 h. After the reaction, the mixture was cooled and centrifuged at 6000 rpm (corresponding to approx. 3100 × g) for 8 min using an IFuge D06 Premium Edition centrifuge (JP Selecta, Barcelona, Spain). The NC was thoroughly washed several times with distilled water, centrifuged, and then dried at 60 °C for 24 h. The resulting material was labeled as NC-P.

Sulfonation was performed using the oxidation followed by sulfonation method under the conditions described by Rajalaxmi et al.^47^. A similar two-step approach (oxidation and then sulfonation) has been previously applied for cellulose sulfonation by Toy et al.^48^ and Lander et al.^49^. A total of 3 ± 0.001 g of NC was suspended in 100 ml of distilled water. Then, 0.60 g of sodium periodate was added and the mixture was stirred continuously at room temperature for 72 h in the absence of light. For sulfonation, 2 ± 0.001 g of the obtained 2,3-dialdehyde NC was dispersed in 50 ml of distilled water, followed by the addition of 3 g of sodium bisulfite. The reaction mixture was stirred at room temperature for 72 h. After the reaction, the mixture was cooled and centrifuged at 6000 rpm (corresponding to approx. 3100 × g) for 8 min using an IFuge D06 Premium Edition centrifuge (JP Selecta, Barcelona, Spain). The obtained NC was washed multiple times with distilled water, centrifuged, and dried at 60 °C for 24 h. The resulting material was labeled as NC-S.

**Fig. 1:**
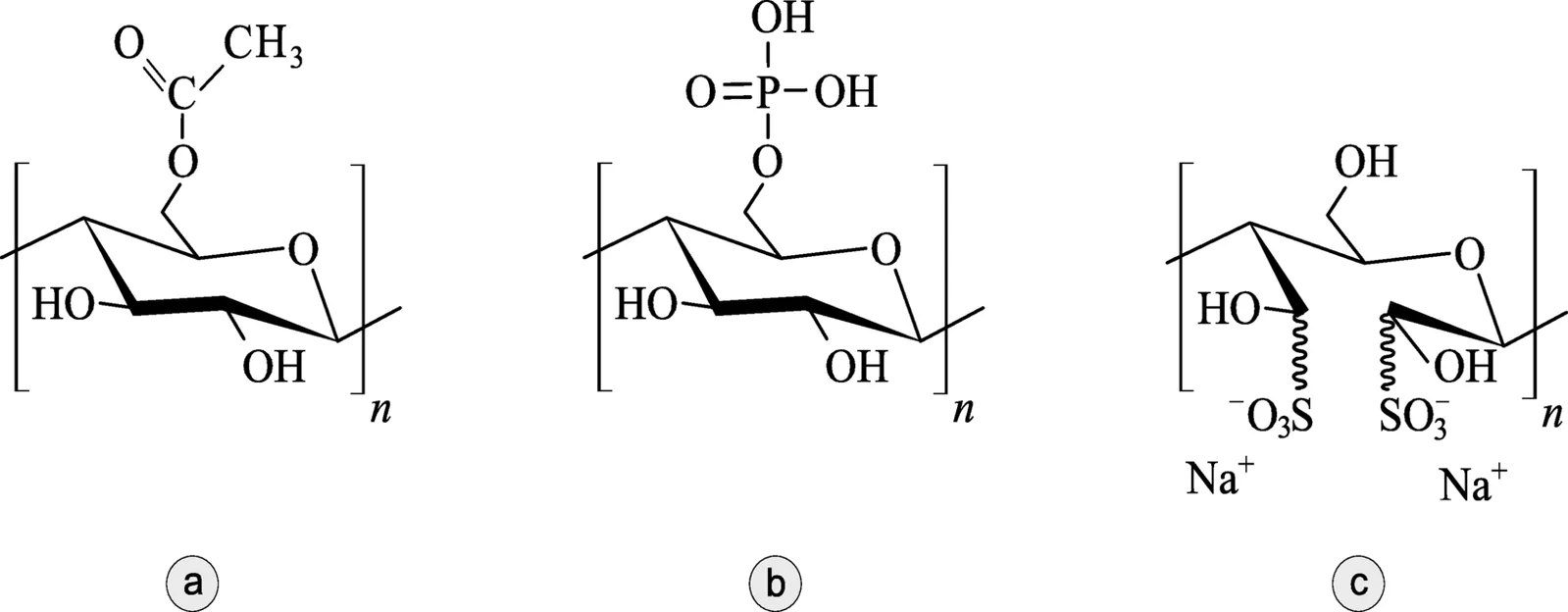
Schematic presentation of: (**a**) acetylated NC; (**b**) phosphorylated NC; (**c**) sulfonated NC.

A schematic presentation of NC after the applied chemical modifications, including acetylation, phosphorylation and sulfonation, is shown in Fig. 1.

### Nanocellulose characterization

To evaluate the effectiveness of the applied modifications, attenuated total reflectance Fourier transform infrared spectroscopy (ATR-FTIR) was conducted using a Nicolet iS5 spectrophotometer (Thermo Fisher Scientific, Waltham, MA, USA) equipped with a deuterated triglycine sulfate detector.

The quantitative determination of acetyl groups was performed in triplicate using the method described by Kim et al.^50^ to confirm the effectiveness of the acetylation. A 100 mg sample of NC-A was placed into 40 ml of 75% ethanol in a glass bottle. The bottle was heated to 55 °C for 30 min while stirring to enhance swelling of the material. Then, 40 ml of 0.5 N NaOH solution was added to the sample, and the mixture was heated to 55 °C for 15 min with continuous stirring. Afterwards, the bottle was tightly sealed and allowed to stand at room temperature for 48 h. Finally, the excess alkali was titrated with 0.5 N HCl using phenolphthalein as an indicator.

To confirm the effectiveness of the phosphorylation, the phosphate group content was determined following the approach described by Liu et al.^51^ and Mautner et al.^52^. Potentiometric titrations were carried out on 0.5 wt% aqueous suspensions of NC‑P (prepared in Milli‑Q water with low carbonate content of < 10^− 5^ mol/l) in a total volume of 50 mL using a Metrohm 902 Titrando system (Metrohm AG, Herisau, Switzerland). The ionic strength was adjusted to 0.1 mol/l with KCl. Before titration, suspensions were ultrasonicated and magnetically stirred for 2 min to ensure homogenous dispersion. All titrations were performed under an inert nitrogen atmosphere to prevent CO_2_ contamination. Titrants used were standardized 0.1 M NaOH and 0.1 M HCl, delivered at a rate of 0.1 ml/min. The pH was measured with a calibrated Metrohm combined glass electrode until a stable reading was obtained after each addition. A blank titration (without NC‑P) was run under identical conditions and the resulting volume was subtracted from sample titrations to correct for background. All measurements were performed in triplicate, and the average phosphate groups content (mmol/g) and standard deviation were reported.

The effectiveness of the sulfonation process was confirmed by determining the sulfur content in NC-S, as in the case of recent work of Toy et al.^48^. The measurements were conducted in triplicate using an EA-1108 elemental analyser (Fisons Instruments, Milan, Italy).

Hydrophilicity of the non-modified and functionalized nanocellulose was assessed based on contact angle (CA) measurements^53^. A 4.0 ± 0.1 mg sample of each variant was pressed into pellets using a manual pellet press (Specac, Warsaw, Poland). The pellets were conditioned for 72 h in a climatic chamber at 20 ± 1 °C and 65 ± 1% relative humidity prior to testing. The contact angle was measured with a DSA25 Krüss goniometer (Merazet, Poznań, Poland) by placing a 4 µL drop of distilled water on the pellet surface. Three replicates were performed for each variant.

### UF adhesive modification and characterization

The nanoparticles were introduced as a 10% aqueous suspension to facilitate homogenization within the adhesive. The amount of suspension added was adjusted to achieve a final NC content of 1%, which was considered optimal based on previous studies^12,15^. In the case of resins intended for particleboard production, high loadings of NC should be avoided due to dispersion difficulties and the risk of excessive viscosity increase, which could potentially reduce adhesive distribution on the surface of the wood particles. Previous studies have shown that a 0.5% addition resulted in a weaker effect compared to 1%, while increasing the loading to 1.5% and 2% did not lead to further significant improvements. Four variants of modified mixtures were prepared, containing non-functionalized, acetylated, phosphorylated, and sulfonated NC. Additionally, a reference variant (REF), consisting of the adhesive mixture without nanoparticles, was used for comparison. Moreover, 2% of 20% aqueous solution of ammonium nitrate was added as a hardener. All components were slowly homogenized at 1000 rpm for approx. 2 min using a CAT-500 homogenizer (Ingenieurbüro CAT, M. Zipper GmbH, Ballrechten-Dottingen, Germany).

The effect on the properties of liquid adhesives was evaluated based on parameters commonly used in industrial quality control of resins in wood-based panel production, such as viscosity, pH, and gel time. Viscosity was measured using a Brookfield DV-II + Pro rotational viscometer (Middleboro, MA, USA) with spindle No. 5, while pH was determined with a Testo 206 pH meter (Pruszków, Poland). Gel time was assessed according to the Polish standard PN-C-89352-3 (1996). Briefly, 10 ± 0.01 g of the adhesive mixture was placed in a test tube and immersed in a water bath at 100 °C. The gel time was recorded as the duration needed for the adhesive to lose its fluidity. All properties were measured in triplicate. The formaldehyde content in the cured adhesive was determined in triplicate according to EN 120 (2011), with modifications based on the method described by Dziurka and Mirski^54^. A 5 ± 0.01 g sample of the ground cured adhesive was extracted using boiling toluene in a perforator apparatus. The formaldehyde concentration was then measured spectrophotometrically at 412 nm using the ammonium acetate and acetylacetone method, with a Biosens UV-5600 spectrophotometer (Biosens, Warsaw, Poland). The thermal stability of the cured adhesive mixtures was evaluated using thermogravimetric analysis (TGA). It was conducted using a TGA4000 thermogravimeter (Perkin Elmer, Waltham, MA, USA) with the temperature raising from room temperature up to 600 °C at a heating rate of 10 °C/min in a nitrogen atmosphere (flow rate 20 ml/min).

### Particleboard manufacturing and characterization

Pine wood particles, dried to a moisture content of 3 ± 2%, were bonded with a gluing degree of 8% in a low-speed rotary gluing machine equipped with a pneumatic spraying system. Two single-layer particleboards (380 mm × 670 mm, 12 mm thickness, target density of 650 kg/m^3^) were produced for each adhesive variant. The pressing process was carried out using laboratory hydraulic press (Siempelkamp GmbH & Co. KG, Krefeld, Germany), under the following conditions: temperature of 160 °C, unit pressure of 2.5 N/mm^2^, and a pressing time of 20 s/mm of final board thickness.

The manufactured particleboards were conditioned for 7 days at 21 ± 2 °C and 65 ± 2% relative humidity prior to testing. Board density was determined in accordance with EN 323 (2001). Mechanical properties were evaluated using 12 samples per variant. Internal bond (IB) was determined according to EN 319 (1993), while bending strength (MOR) and modulus of elasticity (MOE) were assessed according to EN 310 (1999). Water resistance after 2 and 24 h of soaking was also evaluated using 12 samples per variant. Thickness swelling (TS) was measured in accordance with EN 317 (1998), and water absorption (WA) was calculated based on the weight difference before and after immersion. Formaldehyde content (FC) was determined in triplicate using the perforator method according to EN 120 (2011). The concentration of formaldehyde in a collected solution was measured spectrophotometrically at 412 nm using the ammonium acetate and acetylacetone method with a Biosens UV-5600 spectrophotometer (Biosens, Warsaw, Poland).

### Statistical analysis

Results are expressed as mean ± standard deviation (SD). Statistical differences between the variants in terms of liquid adhesive properties, formaldehyde content in cured adhesive, and particleboard properties were evaluated using one-way analysis of variance (ANOVA), followed by Tukey’s Honestly Significant Difference (HSD) post hoc test. Differences were considered statistically significant at *p*-value < 0.05.

## Results and discussion

### Characterization of modified nanocellulose

**Fig. 2:**
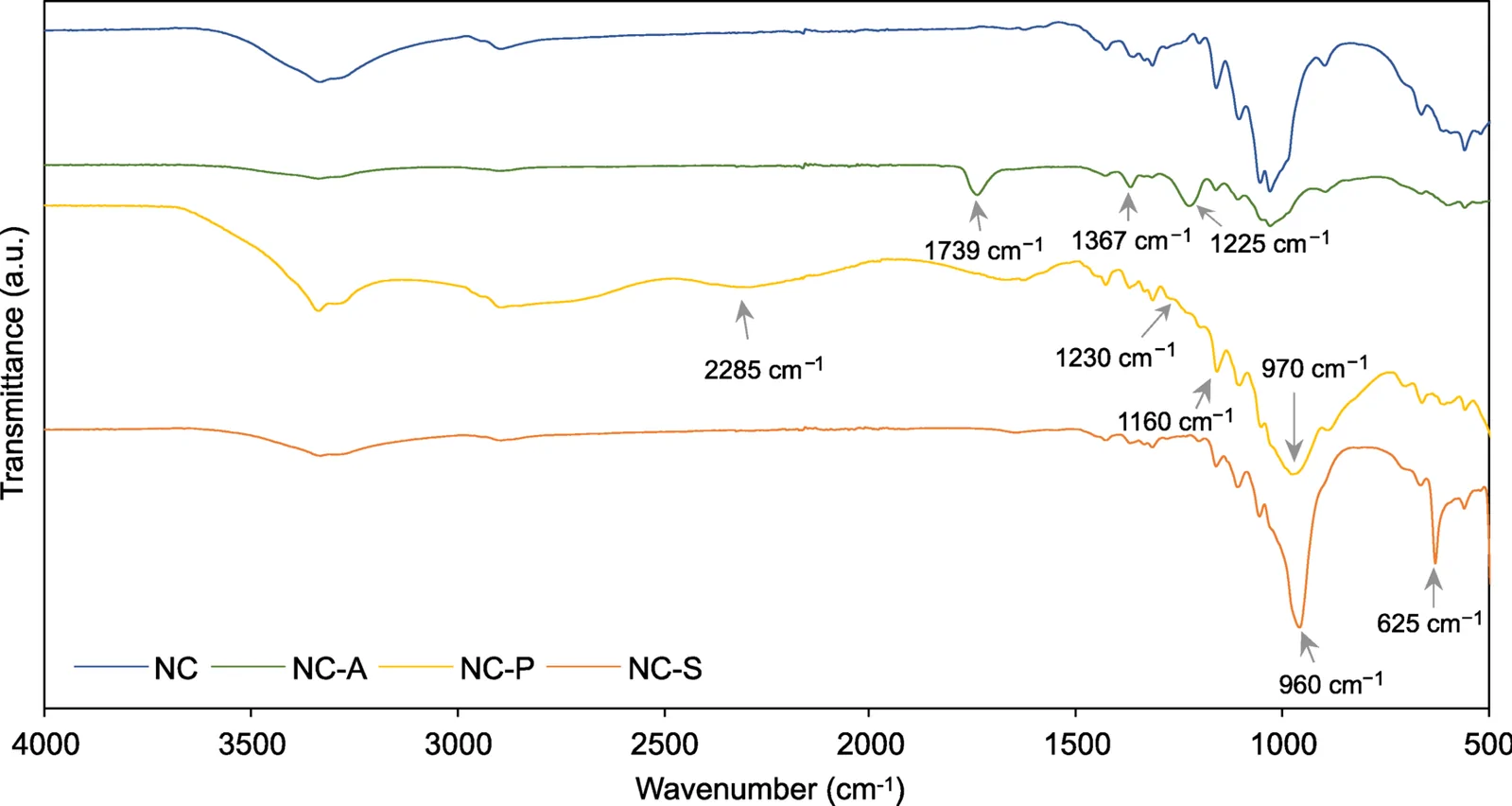
Spectra of unmodified and modified NC.

As shown in Fig. 2, the ATR-FTIR spectra of NC revealed typical cellulose bands observed at 3327 cm^− 1^ (O − H stretching vibrations), 2890 cm^− 1^ (C − H stretching vibration), 1315 cm^− 1^ (C−H_2_ wagging vibration), 1161 cm^− 1^ (bridge oxygen C − O stretching vibration), 1054 cm^− 1^ (C − H stretching vibration), 1027 cm^− 1^ (C − O−C stretching vibration), and 894 cm^− 1^ (C − O stretching vibration)^26,55,56^. In the spectrum of NC-A, three additional bands were observed. Their presence was the evidence of an effective acetylation reaction. These bands appeared at 1739 cm^− 1^ (stretching vibration of C = O of acetyl groups), 1367 cm^− 1^ (stretching vibration of C − H in − O(C = O)−CH_3_) and 1225 cm^− 1^ (stretching vibration of C − O of acetyl groups)^57,58^. Moreover, reduced intensity of − OH stretching band occurring in the range around 3327 cm^− 1^ due to the replacement of O − H groups with acetyl groups is the additional evidence of effective acetylation^57^. Furthermore, in the spectrum of NC-A, the absence of bands in the region between 1840 and 1760 cm^− 1^_,_ and at 1700 cm^− 1^ indicated that it was free from unreacted acetic anhydride^59^. The spectrum of NC-P contained additional bands at around 2285 cm^− 1^ (P − H stretching vibration), 1230 cm^− 1^ (P = O stretching vibration), 1160 cm^− 1^ (C − O−P vibration mode), and several bands between 1100 and 880 cm^− 1^ (P − OH stretching vibrations), the presence of which confirmed the effectiveness of the phosphorylation^60,61^. Furthermore, the spectrum of the NC-S showed additional bands which may confirm structural changes in the nanocellulose after performed modification. These bands were observed at 1025 cm^− 1^ and 958 cm^− 1^, which can be attributed to S − O stretching vibrations, and bands in the region of 495–728 cm^− 1^, which can be attributed to S = O stretching vibrations, indicating successful sulfonation^49,62,63^.

In addition to ATR-FTIR, further analyses were carried out using quantitative chemical analysis methods, which are often applied as a complement to spectroscopic techniques. The acetyl group content in NC-A was quantified at 1.012 ± 0.011 mmol/g which confirms the effectiveness of acetylation. The phosphate group content in NC-P reached 0.433 ± 0.013 mmol/g which indicate successful phosphorylation. Furthermore, the sulfur content in NC-S was 0.21 ± 0.02%, indicating the effectiveness of the sulfonation process. Analyses of the unmodified NC did not detect acetyl or phosphate groups and sulfur, at levels above the detection limits.

The CA measurements showed significant changes in the surface hydrophilicity of the modified nanocelluloses (Table S2). Phosphorylation and sulfonation decreased the CA from 63.1° for unmodified NC to 35.2° and 34.7° for NC-P and NC-S, respectively, corresponding to a reduction of approx. 44–45%. This decrease may be attributed to the introduction of hydrophilic phosphate and sulfonic moieties on the nanocellulose surface, which is consistent with previously reported results^64,65^. In contrast, acetylation increased the CA to 76.5° for NC-A, representing an increase of about 21% compared to unmodified NC. This enhancement in hydrophobicity was expected, as acetylation of nanocellulose with acetic anhydride is recognized as one of the most effective methods to introduce hydrophobic moieties onto the surface of NC^66^.

### Properties of adhesive mixtures

The properties of the adhesive mixtures are presented in Table 1. Based on the results, it was found that both the addition of unmodified NC and its functionalization had a statistically significant effect on properties such as viscosity and gel time, while no significant effect was observed on pH and formaldehyde content. The addition of NC resulted in an increase in the viscosity of the mixtures, most likely due to its strongly hydrophilic nature and high affinity for water. Acetylation of NC did not show a significant effect compared to unmodified NC. However, both phosphorylation and sulfonation caused a further, statistically significant increase in viscosity of adhesive mixtures. The rise observed in the case of formulations containing NC-P and NC-S may be attributed to the increased hydrophilicity of NC caused by these modifications, electrostatic repulsion and network formation. Overall, the viscosity of all mixture variants fell within the range between 100 and 450 mPa·s, which Pizzi & Mittal^67^ described as suitable for particleboard production. Furthermore, it has been shown that the addition of NC shortens the gel time of adhesive mixtures, which may be related to the large specific surface area of NC and the availability of hydroxyl groups facilitating the formation of hydrogen bonds between the nanoparticles and the UF resin. Among the applied modifications, only acetylation caused a statistically significant reduction in gel time compared to unmodified NC. This may indicate improved dispersion considering that Zhang et al.^68^ reported that limited agglomeration of nanoparticles contributes to a decrease in their heat capacity, which consequently enhances the reactivity of the mixture.

**Table 1:** Properties of adhesive mixtures (superscript letters next to the mean values indicate homogeneous groups).

Variant	Viscosity (mPa·s)	Gel time (s)	pH	Formaldehyde content (mg/100 g)
REF	212.6^a^ ± 7.3	112^c^ ± 2	6.46^a^ ± 0.05	65.39^a^ ± 0.26
NC	249.3^b^ ± 7.9	106^b^ ± 3	6.45^a^ ± 0.04	65.22^a^ ± 0.22
NC-A	239.7^b^ ± 8.4	97^a^ ± 2	6.48^a^ ± 0.06	65.31^a^ ± 0.31
NC-P	268.3^c^ ± 6.2	104^b^ ± 2	6.41^a^ ± 0.04	65.17^a^ ± 0.28
NC-S	267.9^c^ ± 7.5	103^b^ ± 3	6.46^a^ ± 0.03	65.27^a^ ± 0.29

**Fig. 3:**
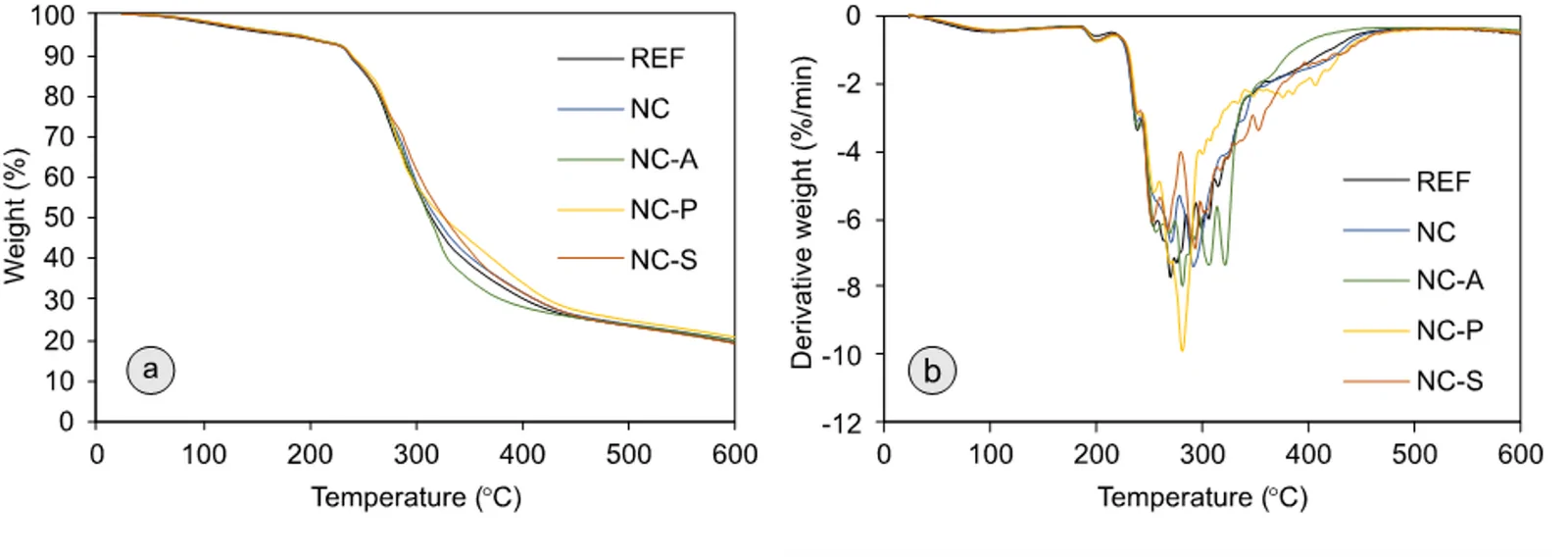
TG (**a**) and DTG (**b**) curves of cured adhesive mixtures.

The thermograms presented in Fig. 3 exhibited a typical thermal decomposition pattern for cured UF resin. The first stage corresponded to a slow weight loss between 25 and 220 °C, which resulted from the removal of water and free formaldehyde from the resin. The second stage, occurring between 220 and 400 °C, was characterized by rapid decomposition and the greatest mass loss resulting from the breakdown of methylene and methylene ether bonds. The third stage occurred above 400 °C and was associated with further decomposition and pyrolysis of the resin and its residues^69^. Detailed thermal parameters obtained from TGA, including the temperatures corresponding to 5%, 10%, 20%, 50 and 70% mass loss (T_5_, T_10_, T_20_, T_50_, and T_70_) as well as the residue at 600 °C, are presented in Table 2. It was shown that the UF resin containing unmodified NC exhibited slightly better thermal stability than pure UF, which can be attributed to hydrogen bonding interactions between NC and the resin matrix that promote the formation of the cross-linking network^70^. Furthermore, no significant effect of NC sulfonation was observed when compared to the variant containing unmodified NC. The effect of NC acetylation on thermal characteristic of UF adhesive was complex. At low temperatures up to approx. 250 °C, the highest thermal stability was observed. Beyond this point, degradation accelerated rapidly, and at high temperatures above 300 °C, it was the lowest among all tested variants. This was most likely related to the fact that acetylated NC decomposes above 250 °C, releasing CO, CO_2_, and acetic acid^71^. According to Roffael & Hüster^72^, thermo-hydrolytic degradation of cured UF resin may be significantly accelerated by the release of acetic acid, which is consistent with the observed effect. As stated by Fu et al.^73^, releasing acetic acid may promote the degradation of cured UF resin by lowering the activation energy necessary for the breakdown of its cross-linked network. The mixture containing NC-P exhibited the best thermal properties, demonstrating high thermal stability at both low and high temperatures, as well as slightly greater residue after the analysis. It was observed that the temperatures corresponding to 50% and 70% mass loss (T_50_ and T_70_) were higher for the adhesive mixture containing NC-P compared to the reference variant by 4% and 6%, respectively. This effect can be attributed to the enhanced thermal stability of NC and promoted char formation caused by phosphorylation^74^.

**Table 2:** Thermal decomposition parameters.

Variant	T_5_ (°C)	T_10_ (°C)	T_20_ (°C)	T_50_ (°C)	T_70_ (°C)	Residue (%)
REF	171.69	237.30	265.14	314.96	401.75	19.2
NC	179.07	237.75	266.39	319.44	411.66	19.9
NC-A	186.91	239.62	266.37	312.73	379.85	19.2
NC-P	183.16	238.75	268.87	326.81	423.46	20.7
NC-S	179.59	238.43	266.92	326.95	412.07	19.9

### Properties of particleboards

**Fig. 4:**
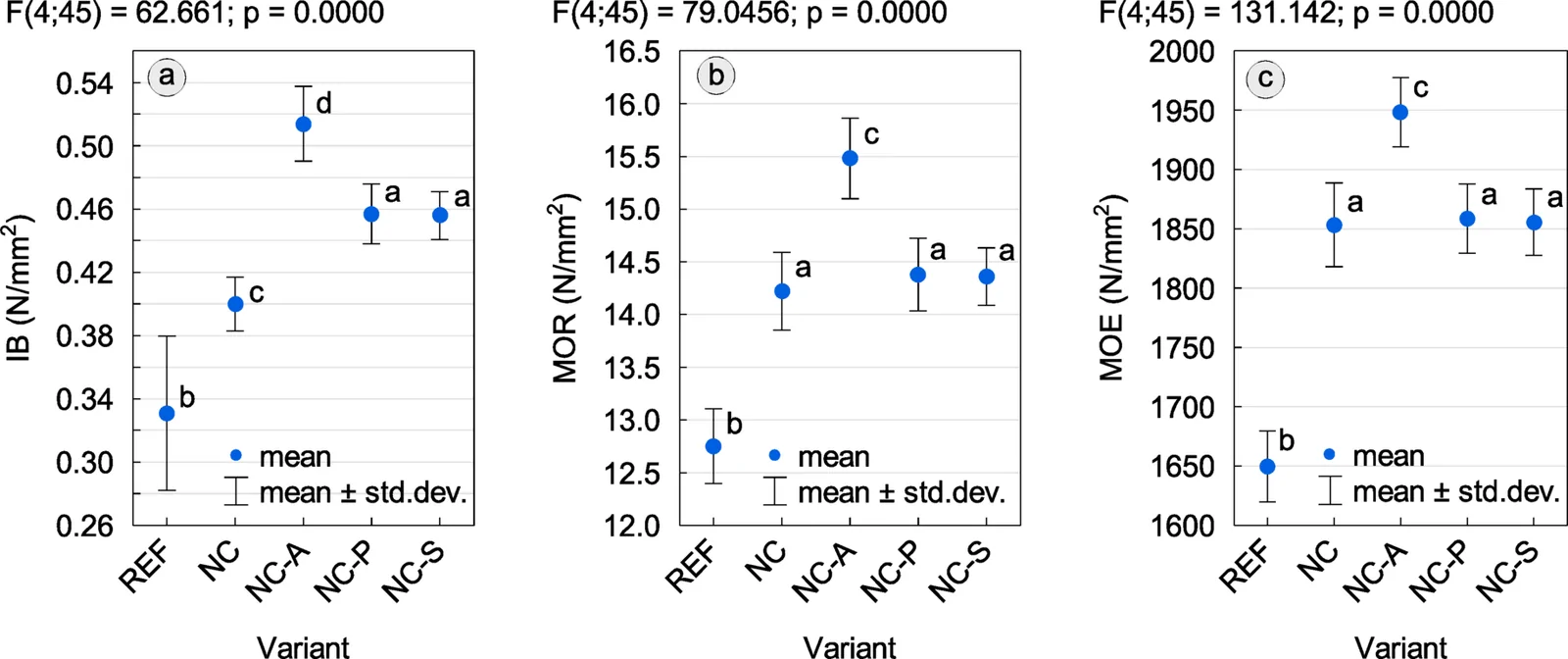
Mechanical properties of particleboards: (**a**) internal bond; (**b**) bending strength; (**c**) modulus of elasticity.

The results showed that reinforcing the resin with both unmodified and modified NC did not cause statistically significant changes in particleboard density (Figure S2). However, the use of the modified adhesive mixtures resulted in a considerable increase in IB (Fig. 4a), which is a fundamental indicator of the binding agents’ performance in wood-based boards. The addition of unmodified NC resulted in a statistically significant increase in IB of approximately 21% compared to the REF variant. This effect can be attributed to a combination of enhanced resin reactivity, improved morphology of bond lines, and interactions between the UF matrix and NC, leading to an increased crosslinking density^6,75,76^. Furthermore, the addition of NC to the UF adhesive causes a toughening effect in the joint, making the bond lines more resistant to crack propagation and failure^77^. The addition of NC-P and NC-S resulted in a further statistically significant increase in IB by 39% and 15%, compared with the REF and NC variants, respectively. The introduction of functional groups through phosphorylation and sulfonation probably enhanced the chemical compatibility of NC with the UF resin which promoted stronger interactions with the matrix. The most pronounced improvement was observed with the addition of NC-A, where IB increased by 54% compared to REF and by 28% compared to NC. According to Sridhar et al.^78^, acetylation leads to a reduction in the number of hydrogen bonds formed between the cellulose surface and liquids. Reduced surface polarity also indicates decreased hydrogen bonding between adjacent nanoparticles, which reduces their agglomeration, and consequently, contributed to the most effective UF adhesive matrix reinforcement through improved NC dispersion^79^.

The results of MOR and MOE are presented in Fig. 4b and c. It was found that the addition of NC, NC-P, and NC-S caused a statistically significant improvement of approx. 12% in both MOR and MOE. Therefore, no effect of phosphorylation and sulfonation on these properties was observed. The improvement was most likely due to the overall enhancement in bond line strength shown by the increase in IB. The best results were obtained with the addition of NC-A, where MOR increased by 21% and MOE by 18% compared to the REF variant. This indicates that only acetylation of NC led to a sufficiently significant improvement in bonding quality to affect the flexural characteristics of particleboard. Moreover, considering that NC acts as a carrier of stresses in the UF matrix, the observed improvement may also indicate better dispersion of the nanoparticles. With limited agglomerate formation, stresses are not concentrated at certain points and are transferred more efficiently within the bond line, allowing the joints to better sustain the load during bending.

**Fig. 5:**
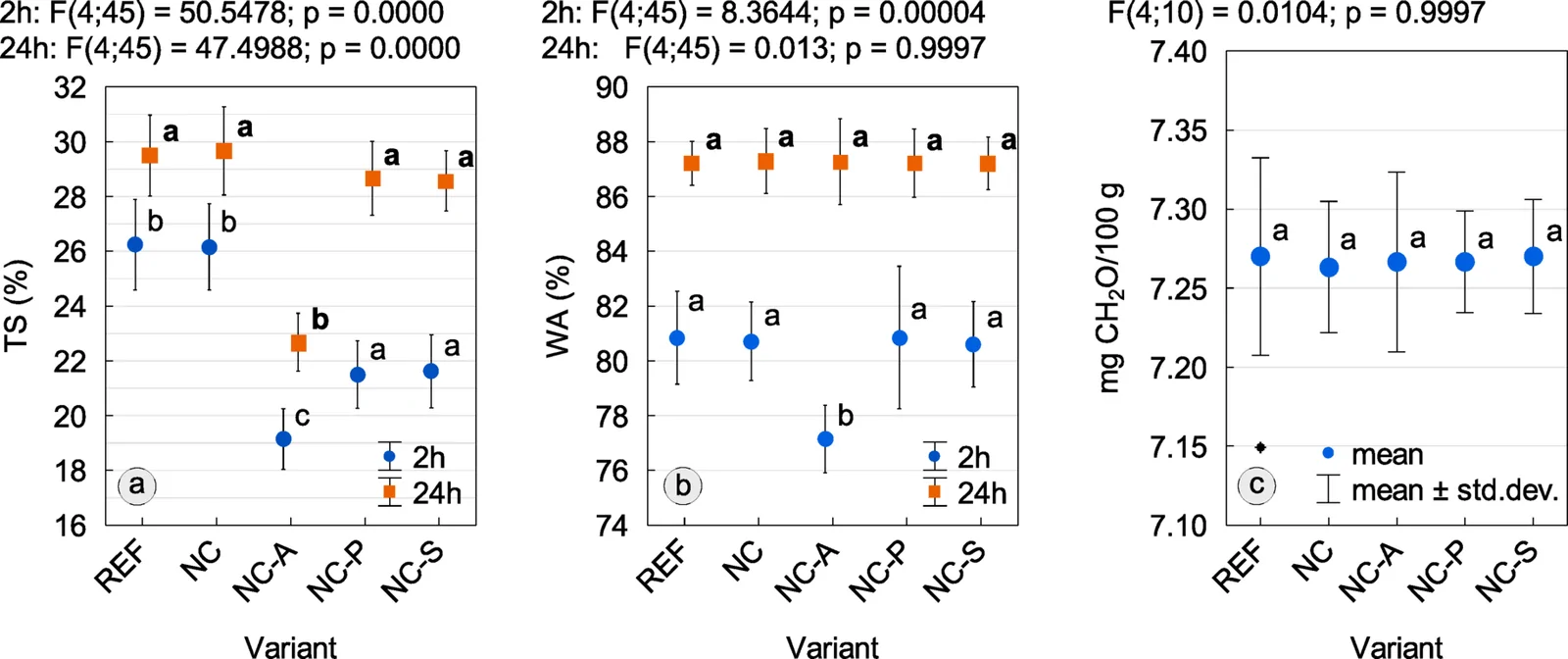
Properties of particleboards: (**a**) thickness swelling; (**b**) water absorption; (**c**) formaldehyde content.

Figure 5a presents the TS results of the manufactured particleboards. The outcomes showed that the addition of unmodified NC did not significantly affect the thickness swelling after both 2 and 24 h of soaking in water. Notably, a positive effect of NC functionalization on this parameter was observed. The addition of NC-P and NC-S reduced TS after 2 h of water immersion by 18%, which was most likely associated with increase in the strength of the adhesive bonds. However, in the case of these variants, 24 h of water immersion was sufficient for degradation to progress, and after this period the changes were no longer statistically significant. It seems that only for the NC-A variant, the increase in bonding strength was sufficient to cause statistically significant changes after both short- and long-term water exposure. In this case, TS was reduced by 27% and 23% after 2 and 24 h of water immersion, respectively. Therefore, the high bond strength most likely made the particleboard more resistant to the swelling stresses occurring during water exposure^80^.

The WA results after 2 and 24 h of water immersion are presented in Fig. 5b. No changes were observed with the addition of either unmodified NC or NC that was phosphorylated and sulfonated. The only statistically significant change was a minor reduction in WA by approx. 6% after 2 h of water immersion observed for the NC-A variant. The swelling was considerably lower in this case compared to the other variants, meaning that the particleboard structure remained more compact, which could have slightly delayed water penetration into the material. However, prolonged exposure allowed penetration into the particleboard, enabling the wood to absorb water and saturate the cell walls, as observed in the other variants.

Figure 5c presents the formaldehyde content of the produced particleboards. The results showed that none of the applied modifications of the UF adhesive, regardless of the NC functionalization method, led to a significant reduction in formaldehyde content. Nevertheless, all particleboard variants met the requirement for the E1 emission class (≤ 8 mg/100 g of dry mass of the board).

The effects of acetylated, phosphorylated and sulfonated NC on particleboard properties were compared with previously reported aminated (EDA, APTES) and MDI-modified NC^11,12,15^. Considering IB, all modifications enhanced the performance compared to the unmodified reference variant. Specifically, IB increased by 40–42% for functionalization with EDA, APTES, and MDI, whereas NC-A achieved the highest improvement (54%), followed by NC-P and NC-S (both 39%). For MOR, NC-A improved the value by 21%, which was higher than NC-EDA (12%) and NC-APTES (19%), but slightly lower than NC-MDI (27%). NC-P and NC-S showed the smallest improvements (both 12%). Furthermore, for MOE, NC-A (18%) matched the performance of NC-EDA and NC-MDI (both 18%), exceeded NC-APTES (10%), NC-P and NC-S (both 12%). Regarding TS after 24 h, NC-A reduced swelling by 23%, which was comparable to NC-EDA (21%) and NC-MDI (17%). In contrast, NC-APTES, NC-P, and NC-S showed no significant effect. These results indicate that NC-A provides the most consistent improvement in mechanical properties and dimensional stability, while NC-P and NC-S offer moderate reinforcement effects. Overall, considering both modification cost and process complexity, functionalization with EDA, APTES, and MDI appears to be less practical and less cost-effective than the NC modifications investigated in this study. This is related to factors such as the use of elevated temperatures, organic solvents, and, in the case of EDA, the requirement for an inert atmosphere. In terms of formaldehyde-scavenging ability, only aminated NC showed a significant reduction (NC-APTES reduced content and emission by 33% and NC-EDA by 18%). In contrast, MDI-modified NC, as well as NC-A, NC-P, and NC-S, did not significantly affect formaldehyde content. This difference may be attributed to the presence of reactive amino groups in EDA and APTES, which can react with formaldehyde, whereas the functional groups in NC-A, NC-P, and NC-S show no scavenging capacity. Interestingly, other studies have shown that phosphorylation can enhance the performance of other bio-based modifiers, such as chitin and chitosan, in reducing formaldehyde content in particleboards. However, this effect may also have been influenced by the significantly higher additive loading introduced to the UF resin, as the authors assumed addition levels ranging from 4 to 10%^81,82^. While acetylated NC demonstrated excellent performance in improving particleboard properties, it should be acknowledged that the use of acetic anhydride and concentrated sulfuric acid presents safety and environmental challenges for large-scale production. Industrial implementation of this and the other two modification methods would require careful handling, waste management, and process optimization. In the context of acetylation, alternative “green” methods are constantly being developed, including enzyme-assisted acetylation and the use of solvents such as ionic liquids or deep eutectic solvents^83,84^. Exploring such approaches in the future could provide interesting insights into more sustainable modification strategies.

## Conclusions

The results of this study confirmed the initial hypothesis that acetylation, phosphorylation, and sulfonation of nanocellulose prior to incorporation into UF resin significantly improve the mechanical and physical properties of the resulting particleboards. ATR-FTIR analysis combined with quantitative chemical methods confirmed the successful functionalization of nanocellulose through acetylation, phosphorylation, and sulfonation. The addition of both unmodified and modified NC affected the properties of UF adhesive mixtures, leading to changes in viscosity and gel time, while pH and formaldehyde content remained unaffected. Gel time was reduced in mixtures containing acetylated NC, whereas viscosity increased most notably in mixtures with phosphorylated and sulfonated NC. Thermal analysis indicated that NC generally improved the thermal stability of UF resin, with phosphorylated NC providing the most stable performance across the temperature range. Variant containing acetylated NC exhibited higher stability at lower temperatures but accelerated degradation at higher temperatures, while sulfonation did not significantly affect thermal stability compared to unmodified NC. Mechanical testing demonstrated that reinforcement of UF adhesive with NC improved the properties of particleboards. Acetylated NC provided the greatest enhancement in IB and was the only variant among those containing functionalized NC to significantly improve flexural properties, including both MOR and MOE. Moreover, functionalized NC improved the dimensional stability of particleboards, with acetylated NC showing the most pronounced effect by reducing thickness swelling after both short- and long-term exposure and limiting water absorption after short-term exposure. Phosphorylation and sulfonation contributed to a reduction in thickness swelling only during short-term exposure. However, none of the NC modifications significantly influenced formaldehyde content. Overall, among the functionalization methods tested, acetylation was identified as the most effective NC modification in this study. It reduced the gel time of UF adhesive, improved thermal stability in the temperature range relevant to particleboard manufacturing and use, and enhanced particleboard performance by improving bonding quality, mechanical properties, and dimensional stability, demonstrating the potential of acetylated NC as a promising reinforcement approach.

## Supplementary Information

Below is the link to the electronic supplementary material.

Supplementary Material 1

## Data Availability

Data will be made available upon request.
