# Supplementary material for: Acetylated, phosphorylated and sulfonated nanocellulose for the modification of urea-formaldehyde adhesive in particleboard production
Source: Sci Rep. 2026 Jun 8;16:26084. doi: 10.1038/s41598-026-56716-x (PMC13490584; doi:10.1038/s41598-026-56716-x)
Supplement: Supplementary file 1 [file 41598_2026_56716_MOESM1_ESM.docx]

**Figure S1.** Fractional composition of wood particles

**Table S1.** Average dimensions and shape factors of wood particles

| **Parameter** | **Formula** | **Value** |
| --- | --- | --- |
| Length (mm) | - | 16.91 ± 4.21 |
| Width (mm) | - | 2.97 ± 1.09 |
| Thickness (mm) | - | 1.14 ± 0.31 |
| Degree of slenderness (λ) | $\lambda=\frac{Mean length}{Mean thickness}$ | 14.83 |
| Degree of flatness (Ψ) | $\Psi=\frac{Mean width}{Mean thickness}$ | 2.61 |
| Width coefficient (m) | $m=\frac{Mean length}{Mean width}$ | 5.69 |

**Table S2.** Contact angles of nanocellulose

| **Variant** | **Contact angle (°)** |
| --- | --- |
| NC | 63.1^b^ ± 0.7 |
| NC-A | 76.5^c^ ± 0.4 |
| NC-P | 35.2^a^ ± 1.1 |
| NC-S | 34.7^a^ ± 0.9 |

**Figure S2**. Density of manufactured particleboards
